# Differential scanning calorimetric investigations of three rotary nickel-titanium instrument systems before and after simulated clinical uses

**DOI:** 10.1186/s12903-021-01857-w

**Published:** 2021-10-02

**Authors:** Khoa Van Pham

**Affiliations:** Department of Operative Dentistry and Endodontics, Faculty of Odonto-Stomatology, University of Medicine and Pharmacy at Ho Chi Minh City, Ho Chi Minh City, 700000 Vietnam

**Keywords:** DSC, Nickel-titanium, Rotary, Root canal, Endodontics

## Abstract

**Background:**

The transformation temperatures were important values, influencing the mechanical properties and clinical performance of nickel-titanium instruments. The aim of this study was to determine the transformation temperatures of three rotary nickel-titanium (NiTi) instruments: Reciproc, HyFlex CM Pro, and Neoniti before and after simulated clinical uses.

**Methods:**

Ninety new NiTi instruments of three single-file instruments: Reciproc, HyFlex CM Pro, and Neoniti were divided into three groups. Thirty instruments of each group were divided into 3 subgroups (10 instruments for each subgroup): new, one-time simulated clinical used and sterilised, and three times simulated clinical used and sterilized subgroups. The instruments were in the as-received condition for the new subgroups, one time used in the plastic endo-training blocks and sterilised for the one-time subgroups, and three times used in the plastic endo-training blocks and sterilised for the three times subgroups. Each instrument in subgroups was cut into four small segments of 4–5 mm. All segments of instruments were analysed using Differential Scanning Calorimetry (DSC). Data was collected and analysed using SPSS version 20.0 with ANOVA test or Kruskal–Wallis test at the significant level of 0.05.

**Results:**

There was not significant difference between before and after simulated clinical use with sterilised procedure in three NiTi instrument systems. The austenite-finish (A_f_) temperatures of three instrument systems were higher than that of the human body (37 °C), of these, the A_f_ temperature of Neoniti was highest and that of HyFlex CM Pro was lowest.

**Conclusions:**

The austenite-finish (A_f_) temperatures of three NiTi instruments were higher than that of human body temperature, therefore, material was in the phase transformation from martensite to austenite, gives the instruments more flexibility when used in the clinical situation.

## Background

The NiTi rotary instruments were used more popular in endodontics because of their special characteristics. Since the beginning of the use of NiTi instrument in endodontics [1], the NiTi material was more investigated and improved. Although the conventional NiTi instruments possessed many advanced characteristics, especially super-elasticity, austenite phase of the material at room temperature made the instruments were not enough flexibility in special clinical situations. Material improvement was continuously developed to give the NiTi material better characteristics by many ways to reduce the breakage of instruments in clinical use [2, 3]. Among different improved methods, the most popular approaches were heat treatment, thermomechanical [4], and Electrical Discharge Machining (EDM) process [5].

Reciproc (VDW GmbH, Munich, Germany) was made of M-wire, which was NiTi wire blank was processed by a series of heat treatments [4]. The Reciproc was characterised by special reciprocating rotary and one of the first single-file systems in the world. The instrument cut with a large rotating angle in the counter-clockwise direction, whereas with a smaller rotating angle in the clockwise direction, the instrument released the stress [6].

HyFlex CM (Coltène/Whaledent, Altstätten, Switzerland) was made of controlled-memory wire. The material was processed with a special thermomechanical procedure. With this special process, the memory of the material was controlled, making the instruments extremely flexible without shape memory [4]. This instrument system was characterised by different cross-section designs in varied sizes of instruments, rotated in continuous mode, and can be used as a single-file system [7].

Neoniti (NEOLIX, Châtres-la-Forêt, France) was made of special NiTi material and was produced using EDM process, a new advanced technology [5, 8]. The process characterized by sparks created by electric discharges at high energy and high frequency between the file and the electrode, that made the melting and evaporation of material on specific positions to produce the desired geometry of product. Neoniti was characterised by non-homothetic cross-section, rotated in continuous mode, and can be used as a single-file system [5].

The phase composition of the structure of the NiTi materials was one of the most important factors influenced the mechanical properties of the NiTi instruments in clinical performance [9]. The phase transformation temperatures and enthalpy changes of the NiTi materials were conveniently investigated by Differential Scanning Calorimetry (DSC) [10].

The purpose of this study was to use the DSC to investigate the phase transformations of these three brands of NiTi rotary endodontic instrument systems. The hypothesis was there was no significant difference in transformation temperatures among the experimental subgroups.

## Materials and methods

Ninety new NiTi instruments of three brands: Reciproc R25 25/0.08, HyFlex CM Pro 25/0.06, and Neoniti A1 25/0.08, 25 mm long, were divided into three groups (30 instruments for each group): group 1 (Reciproc), 2 (HyFlex CM Pro), and 3 (Neoniti A1). Each group was divided into 3 subgroups. For Reciproc group, instruments were divided into three subgroups (10 instruments for each subgroup): 1.1 (new), 1.2 (one time simulated clinical used and sterilized), 1.3 (three times simulated clinical used and sterilized), and similar to that for HyFlex CM Pro (2.1, 2.2, 2.3) and for Neoniti A1 (3.1, 3.2, 3.3). The first three subgroups (1.1, 2.1, 3.1) used the new instruments for the cutting step (the following described step). The 60 remaining instruments were used for 19-mm plastic Endo Training Block-L (Dentsply-Maillefer, Ballaigues, Switzerland) instrumentation for the first time and then were sterilized for the first time. The second three subgroups (n = 30) were randomly chosen for the cutting step. The third three subgroups (n = 30) were used for plastic endo blocks instrumentation for the second, third times, and then sterilised for the second, third times, respectively. Finally, these last three subgroups were prepared for the cutting step. For preparing the plastic endo training block-L, instruments were used in the VDW.SILVER®RECIPROC® (VDW GmbH, Munich, Germany) endo motor with the manufacturer’s parameters. Reciproc instruments were used at Reciproc All Mode of the endo motor, HyFlex CM Pro instruments were used at 500 rounds per minute (rpm), torque of 2.5 N.cm, and Neoniti A1 instruments were used at 500 rpm, torque of 1.5 N.cm. Each plastic endo training block was used for only one NiTi instrument and irrigation was performed using 3% sodium hypochlorite (Canal Pro, Coltene Whaledent, Altstätten, Switzerland). All instruments were used in slow in-and-out motion inside the plastic canal until reaching the working length.

Before sterilization, all used instruments were disinfected in 0.5% Hexanious G + R (F.M. Medical, Bénifontain, France) in 15 min. Sterilization parameters were 134 °C in 20 min.

Each instrument in subgroups was cut into four small segments of 4–5 mm (A, B, C, and D from the tip of instrument respectively) using the low-speed saw IsoMet (Buehler, Lake Bluff, IL, USA). All segments of instruments were analysed using differential scanning calorimetry machine DSC 8000 with Pyris software version 11.0.0.0449 (Perkin Elmer, USA). The DSC was performed from 15 to 100 °C. The sample was cooled from room temperature to 15 °C, then heated to 100 °C to receive the heating DSC curve, and subsequently cooled from 100 to 15 °C to receive the cooling DSC curve. Heating or cooling rate was at 10 °C per minute and the speed of nitrogen was at 20 mL per minute. Temperature calibration of the DSC apparatus was conducted with n-pentane, deionized water, and indium. The DSC plots and results were received and analysed by Pyris software included peak temperatures for the phase transformation (start and finish temperatures of phase transformations at endothermic and exothermic peaks on DSC curves), and enthalpy changes related to these processes.

Data was collected and analysed using the SPSS version 16.0 (IBM, Armonk, NY, USA) with the ANOVA test or Kruskal–Wallis test at the significant level of 0.05.

## Results

The austenite-start (A_s_) and austenite-finish (A_f_) temperatures of three NiTi instrument systems were displayed in the Table 1. The A_f_ temperatures of all three brands of instruments were higher than human body temperature.

**Table 1:** The A_s_ and A_f_ temperatures of the three experimental NiTi instrument systems

Subgroups	A_s_ (°C)				A_f_ (°C)			
	Segments							
	A	B	C	D	A	B	C	D
	Mean ± Standard deviation							
1.1	30.69 ± 0.98	31.55 ± 1.09	32.79 ± 1.15	31.97 ± 0.69	47.65 ± 0.48	49.36 ± 0.73	50.62 ± 1.03	51.31 ± 1.79
1.2	30.70 ± 0.81	31.35 ± 1.24	32.95 ± 1.16	31.91 ± 1.12	47.43 ± 0.45	49.45 ± 0.35	50.45 ± 1.08	51.11 ± 1.50
1.3	30.53 ± 0.72	31.47 ± 1.04	32.84 ± 0.98	31.87 ± 0.78	47.62 ± 0.70	49.36 ± 0.46	50.34 ± 1.10	51.12 ± 1.10
*P* value	0.883	0.924	0.945	0.967	0.634	0.904	0.845	0.943
2.1	35.62 ± 0.34	37.06 ± 0.47	38.92 ± 0.56	41.78 ± 0.44	41.45 ± 0.33	42.90 ± 0.75	44.99 ± 0.60	49.57 ± 0.85
2.2	35.51 ± 0.28	37.10 ± 0.43	38.97 ± 0.59	41.81 ± 0.40	41.42 ± 0.40	42.63 ± 0.62	45.10 ± 0.81	50.01 ± 0.83
2.3	35.46 ± 0.29	36.65 ± 0.67	38.73 ± 0.35	41.86 ± 0.33	41.47 ± 0.41	43.48 ± 0.90	45.39 ± 0.66	50.29 ± 0.77
*P *value	0.474	0.136	0.542	0.906*	0.958	0.055	0.438	0.158
3.1	44.57 ± 1.13	43.94 ± 0.55	43.58 ± 0.79	43.98 ± 0.45	53.89 ± 0.83	53.00 ± 1.22	52.37 ± 1.17	53.08 ± 1.11
3.2	44.62 ± 1.22	44.01 ± 0.56	43.77 ± 0.96	43.83 ± 0.68	54.07 ± 1.20	53.39 ± 0.99	52.38 ± 1.34	53.40 ± 1.09
3.3	44.59 ± 0.97	44.08 ± 0.62	43.82 ± 0.59	43.93 ± 0.60	53.79 ± 0.94	53.28 ± 1.11	52.36 ± 1.07	53.18 ± 1.31
*P *value	0.993	0.873	0.792	0.850	0.823	0.718	0.999	0.826

*P* > 0.05, ANOVA test

*Kruskal Wallis test

The R-start (R_s_) and R-finish (R_f_) temperatures of three NiTi instrument systems were displayed in the Table 2. The R_s_ and R_f_ temperatures of the Neoniti A1 instruments were highest in the three brands of instruments.

**Table 2:** The R_s_ and R_f_ temperatures of the three experimental NiTi instrument systems

Subgroups	R_s_ (°C)				R_f_ (°C)			
	Segments							
	A	B	C	D	A	B	C	D
	Mean ± Standard deviation							
1.1	43.48 ± 0.63	43.54 ± 1.18	43.55 ± 1.03	43.83 ± 0.88	26.46 ± 0.20	26.58 ± 0.16	26.79 ± 0.50	26.93 ± 0.68
1.2	43.90 ± 0.84	43.74 ± 0.70	43.55 ± 0.82	43.82 ± 0.82	26.47 ± 0.77	26.58 ± 0.21	26.66 ± 0.29	26.91 ± 0.68
1.3	43.49 ± 0.97	43.64 ± 1.03	43.88 ± 1.18	43.87 ± 0.82	26.44 ± 0.20	26.55 ± 0.18	26.66 ± 0.35	26.93 ± 0.75
2.1	36.19 ± 0.44	37.16 ± 0.20	39.97 ± 0.65	44.78 ± 0.35	30.77 ± 0.37	31.13 ± 0.32	32.83 ± 0.49	35.16 ± 0.51
2.2	36.19 ± 0.47	37.36 ± 0.54	40.12 ± 0.62	44.98 ± 0.47	30.82 ± 0.32	31.06 ± 0.42	32.51 ± 0.43	35.20 ± 0.58
2.3	36.12 ± 0.44	37.49 ± 0.61	40.38 ± 0.40	45.17 ± 0.38	30.76 ± 0.40	31.05 ± 0.44	32.36 ± 0.46	35.38 ± 0.50
3.1	45.32 ± 0.47	45.26 ± 0.62	45.37 ± 0.38	45.39 ± 0.39	36.47 ± 0.63	36.43 ± 0.61	36.16 ± 0.49	35.11 ± 0.78
3.2	45.31 ± 0.44	45.43 ± 0.41	45.37 ± 0.43	45.48 ± 0.42	36.58 ± 1.04	35.96 ± 0.84	36.32 ± 0.54	35.06 ± 1.00
3.3	45.23 ± 0.44	45.31 ± 0.42	45.36 ± 0.30	45.43 ± 0.36	36.31 ± 0.58	36.11 ± 0.57	36.16 ± 0.46	35.03 ± 0.82

The enthalpy changes in the heating and cooling processes were displayed in the Table 3.

**Table 3:** The enthalpy changes in the heating (∆H_1_) and cooling (**∆**H_2_) processes

Subgroups	∆H_1_ (J/g)				∆H_2_ (J/g)			
	Segments							
	A	B	C	D	A	B	C	D
	Mean ± Standard deviation							
1.1	1.51 ± 0.13	1.68 ± 0.13	1.31 ± 0.21	1.67 ± 0.11	1.00 ± 0.23	1.20 ± 0.15	1.28 ± 0.12	1.37 ± 0.12
1.2	1.42 ± 0.11	1.34 ± 0.13	1.33 ± 0.14	1.52 ± 0.13	0.79 ± 0.19	1.01 ± 0.12	1.15 ± 0.17	1.22 ± 0.11
1.3	0.96 ± 0.22	0.96 ± 0.37	1.10 ± 0.29	1.39 ± 0.14	0.63 ± 0.13	0.94 ± 0.13	1.05 ± 0.22	1.19 ± 0.13
2.1	3.74 ± 0.32	4.88 ± 0.31	4.25 ± 0.52	3.72 ± 0.18	1.68 ± 0.25	2.98 ± 0.40	3.44 ± 0.29	3.45 ± 0.13
2.2	0.85 ± 0.15	5.81 ± 0.81	5.18 ± 0.48	4.06 ± 0.15	0.48 ± 0.33	2.97 ± 0.93	3.54 ± 0.33	3.53 ± 0.20
2.3	0.76 ± 0.13	4.36 ± 0.17	4.32 ± 0.10	3.79 ± 0.08	0.40 ± 0.26	2.29 ± 0.51	3.59 ± 0.33	3.41 ± 0.20
3.1	6.58 ± 0.95	7.76 ± 0.65	7.53 ± 1.49	6.26 ± 0.46	2.40 ± 0.37	4.19 ± 0.23	4.54 ± 0.18	4.64 ± 0.20
3.2	6.61 ± 0.49	7.70 ± 1.06	7.65 ± 1.54	6.45 ± 0.69	2.34 ± 0.32	3.99 ± 0.22	4.36 ± 0.22	4.66 ± 0.27
3.3	6.27 ± 0.70	7.71 ± 0.80	7.11 ± 1.06	6.06 ± 0.62	2.08 ± 0.52	3.97 ± 0.21	4.44 ± 0.36	4.65 ± 0.29

**Fig. 1:**
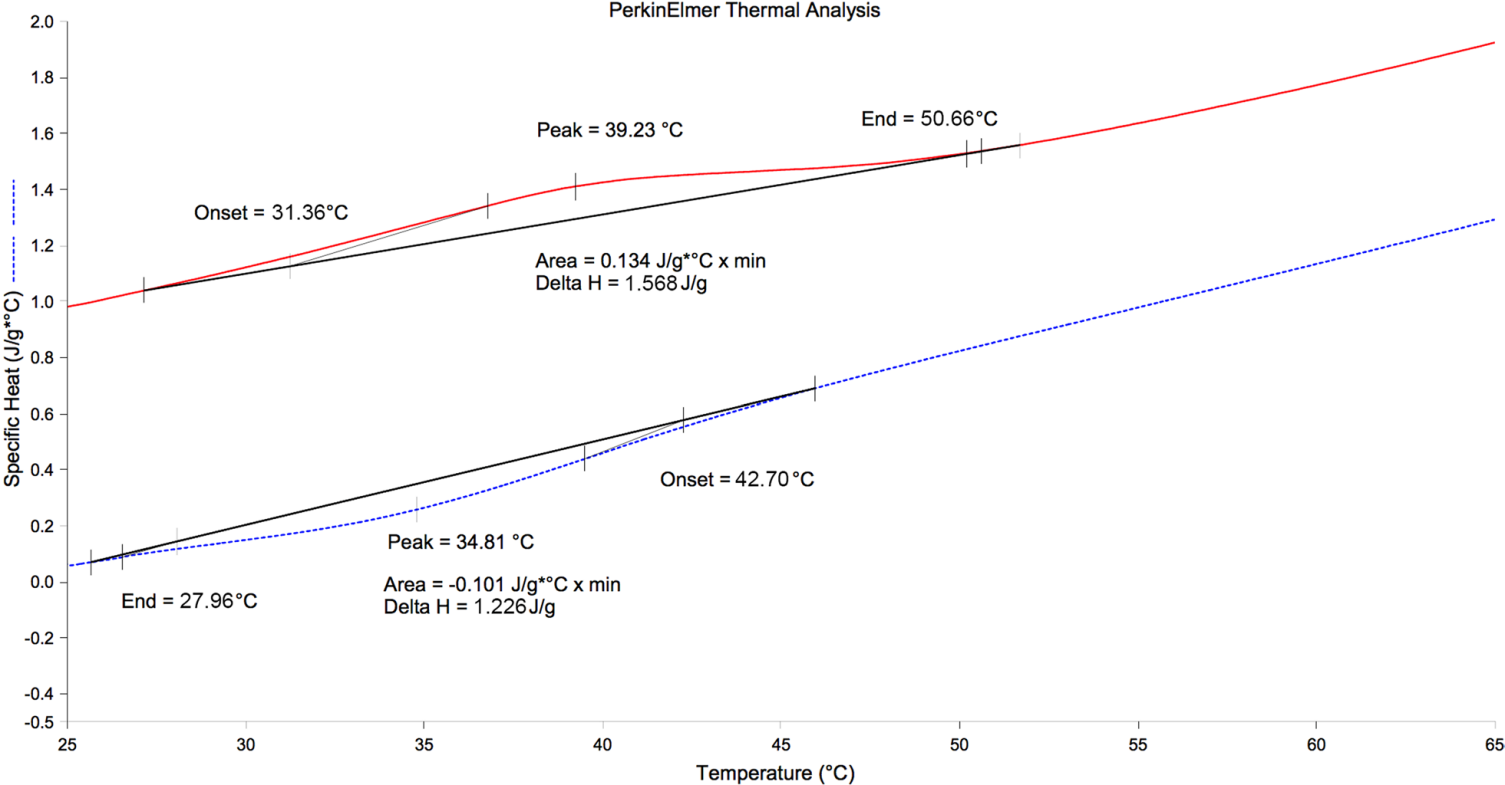
DSC plots of Reciproc (red line: heating curve, blue line: cooling curve)

**Fig. 2:**
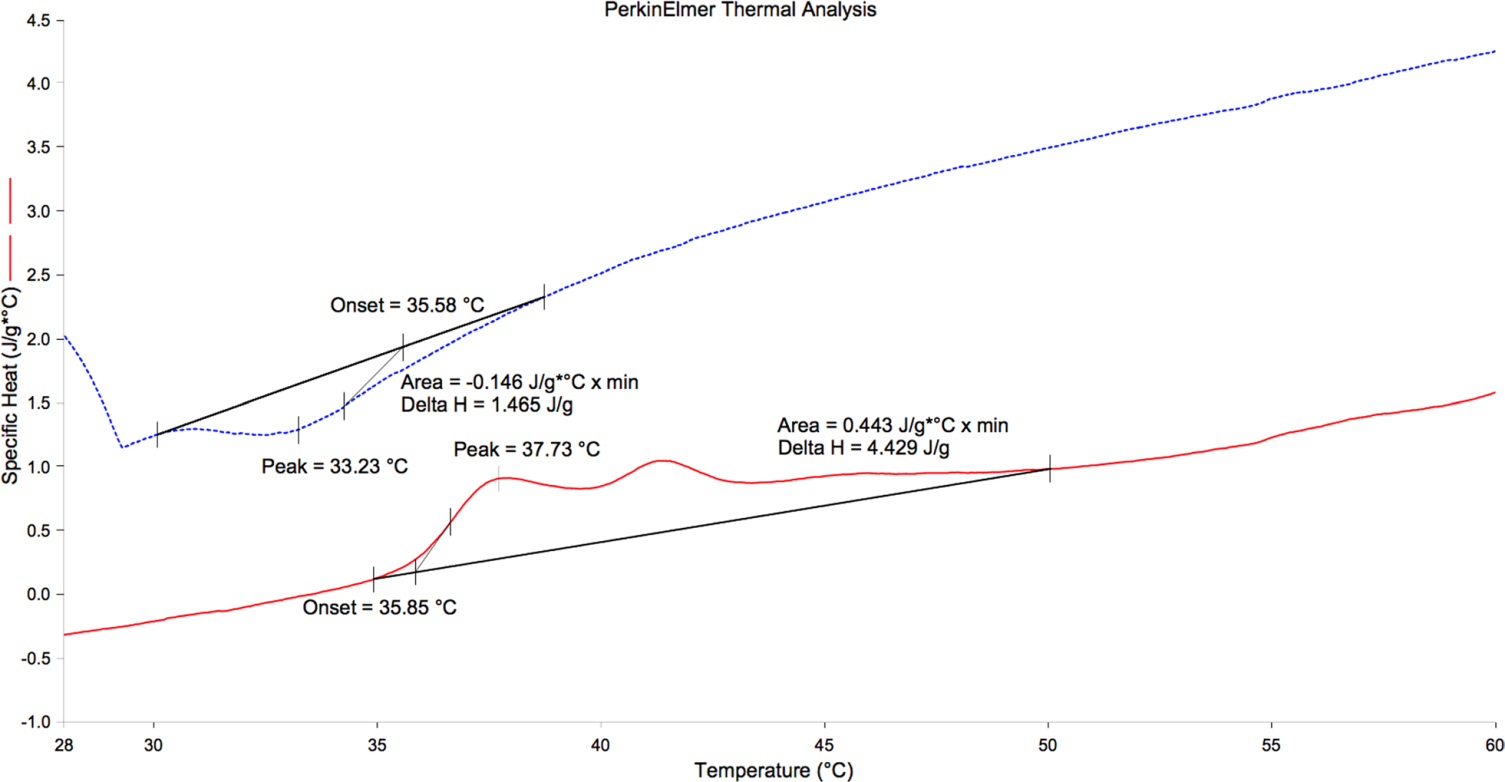
DSC plots of HyFlex CM Pro (red line: heating curve, blue line: cooling curve)

**Fig. 3:**
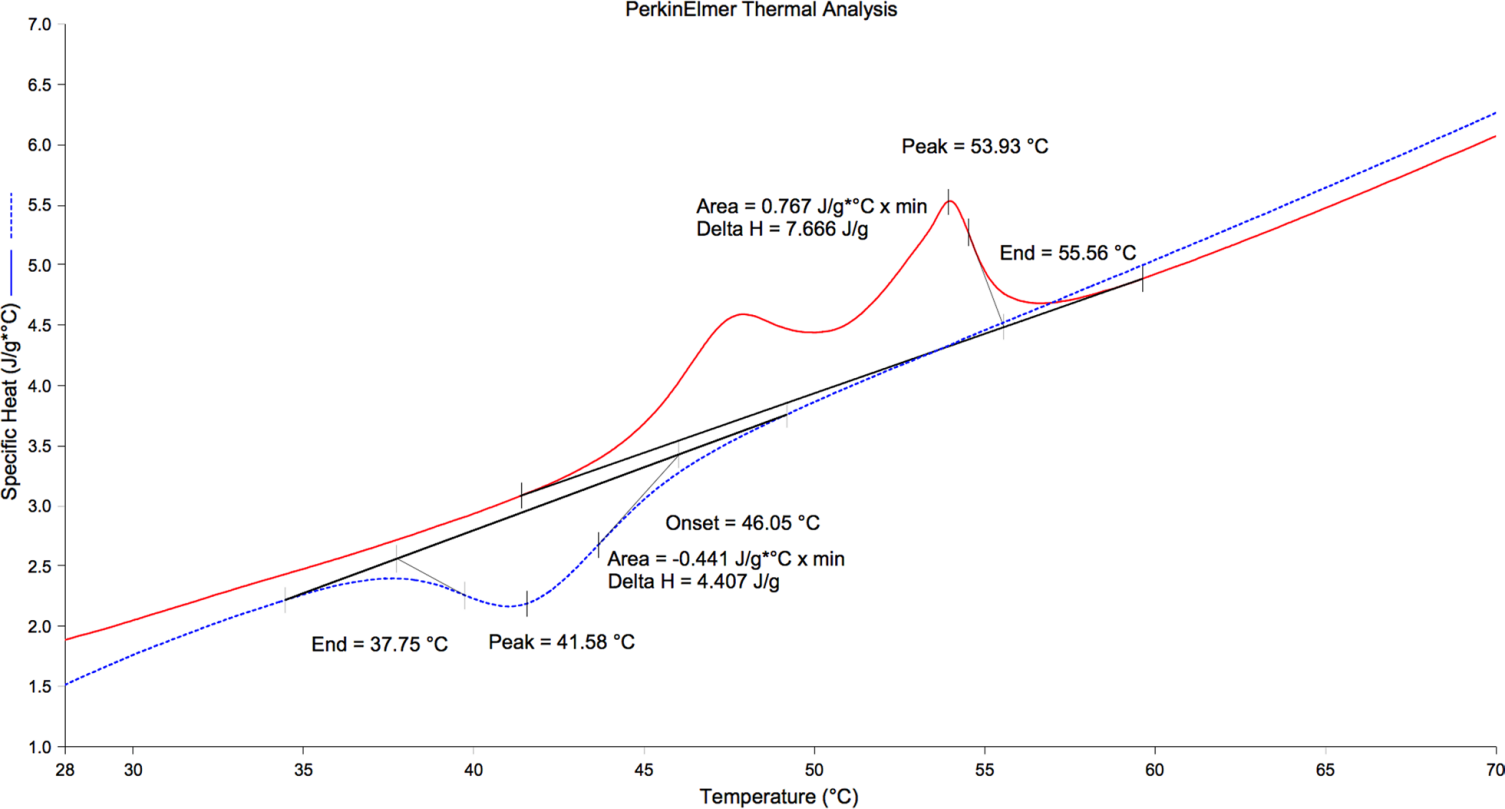
DSC plots of Neoniti (red line: heating curve, blue line: cooling curve)

There was only one endothermic peak on the heating curve and one exothermic peak on the cooling curve for Reciproc M-Wire instrument (Fig. 1). There were two endothermic peaks on the heating curve and one exothermic peak on the cooling curve for HyFlex CM Pro and Neoniti instruments (Fig. 2 and 3).

## Discussion

All three NiTi instruments were used as a single-file technique for root canal preparation. Reciproc was used in reciprocating movement and other two instruments, HyFlex CM Pro and Neoniti, were used in continuing rotary. Reciproc was made of M-Wire, HyFlex CM Pro was produced from controlled memory material and the last one, Neoniti was manufactured using electrical discharge machining (EDM) process [5].

The endo plastic blocks were used popular in investigations root canal preparation, first in 1975 [11]. These endo plastic blocks were produced in similar root canal shapes, lengths, tapers, and curvatures. This eliminated pre-investigation bias that may affect the results of the study. The endo training blocks were used for the simulated clinical preparation because of their hardness and curvature standards when compared to the extracted human teeth. However, the plastic hardness was lower than extracted tooth dentin hardness. Moreover, the friction between the plastic and instrument makes the plastic material to melt, preventing the rotary of the instrument inside the plastic root canal, making the instrument be stuck easily and might cause breakage of instrument. However, there were not any breakage instruments in this study.

Repeated uses of NiTi instruments in clinical were not recommended by many manufacturers nowadays, however other authors reported some NiTi instruments could be used up to ten times in endo plastic blocks or four molars without any breakages [12, 13]. Many authors agreed that long clinical use of these NiTi instruments reduced considerable its fatigue cyclic resistance [14–16]. The rotary instruments were subjected the complexity of the stresses, including the polar moment of inertia, the paradigm shift had been recently introduced, which was considered as the most important cross-sectional factor over other factors such as metal mass and cross-sectional area in determining the torsional resistance of the instruments [17].

Because of different root canal anatomy, other affect factors, it is difficult to recommend about the number of clinical use times for NiTi instruments. In this study, there were unrecoverable unwinding signs of apical parts of HyFlex CM Pro instruments, and it needed more time to reach the working lengths of Reciproc and Neoniti instrumentation after three times of instrumentation and sterilization. Therefore, three times uses were chosen for all these three NiTi instrument systems for this study.

The data (Table 1) showed that the A_f_ temperatures of all three brands of instruments were higher than human body temperature, and this is the advantageous characteristic of these three experiment brands over the other instrument systems made by the conventional NiTi material [10, 18]. The transformation temperatures of the NiTi alloy were dependent on the material composition, and sensitive to the additive of alloying elements [19].

There were no significant differences in the phase transformation temperatures of these three NiTi instruments before and after simulated clinical uses (*P* > 0.05). This result was like that of the previous study [20].

There were significant differences about the R_s_ and R_f_ temperatures of segments A and D of HyFlex CM Pro instruments (Table 2), whereas these values of other two brands’ instruments were similar. These results showed that there were different characteristics of materials that made for the apical and the shaft parts of HyFlex CM Pro instruments. However, this needed to be investigated in future research.

The enthalpy changes for the overall martensitic NiTi to austenitic NiTi transformations (**∆**H_1_) for the new, one and three times simulated clinical uses Neoniti A1 were higher than that for the HyFlex CM Pro and Reciproc (Table 3). These results showed that the amounts of stable, work hardened martensitic NiTi (which does not undergo transformation to austenitic NiTi) in the Neoniti A1 instruments were higher than that in the two latter. Although this suggested that the Neoniti A1 have a more advantageous microstructure, further research needed to be conducted to prove this assumption.

The enthalpy changes (**∆**H_1_ and **∆**H_2_) for segments A of HyFlex CM Pro were higher in the new instruments than that in the one time and three times simulated clinical uses. These results showed that the apical parts of the HyFlex CM Pro were subjected to the simulated clinical stresses and were significantly changed the microstructure. This warned the clinicians to consider the utilization of these instruments for many times in clinical situation. The DSC analysis was the appropriate method to investigate the phase transformations in conventional rotary NiTi instruments [10, 18, 20]. The structure changes in the material could be recognized through the endothermic peaks on the heating curves and exothermic peaks on the cooling curves. The X-ray diffraction (XRD) analysis also was a useful method to investigate the structure of the NiTi alloy [21], however, this technique only evaluated the 50 µm surface structure, whereas the DSC analysis can offer the information about phase transformations, temperature change effects, and enthalpy changes of phase transformations [18, 21].

Heating and cooling rate were the important parameters in DSC analysis. This present study used the heating and cooling rate at 10^0^C per minute, that like the other previous studies [10, 18, 22]. This rate was appropriate for reducing noise signals in the process of DSC analysis.

The working chamber of the DSC apparatus was small, whereas the NiTi instruments were long. Therefore, the instruments should be cut into smaller segments to appropriate with the chamber.

On the DSC plots of these experimental instruments, there was only one endothermic peak on the heating curve and one exothermic peak on the cooling curve for Reciproc M-Wire instrument. Therefore, there was single-stage phase transformation (from austenite to martensite) in Reciproc instrument that experienced under DSC investigation [23]. This showed that there was not any R transformation in the Reciproc instrument under working condition [8]. This result agreed with the finding of the previous study using DSC investigation, which showed that there was also one unique peak on the heating and cooling curve of the ProFile Vortex M-Wire [24]. According to the previous study [20], M-Wire was produced under a special process that made material possessed austenite, martensite and also R-phase with different rates in the instrument. Therefore, it may be understood that under different special conditions of manufacturing, the compositions of M-Wire and ProFile Vortex M-Wire were not identical. Multiple file preparation system HyFlex CM Pro owned two defined peaks on the heating curve and therefore this instrument experienced R-phase transformation in this study. HyFlex CM and HyFlex EDM were also characterized by these features and therefore these instruments had experienced R-phase transformation and contained a certain amount of R-phase in the previous studies using DSC analysis [25, 26]. However, the A_s_ and A_f_ temperatures of the HyFlex CM Pro in the present study were higher than those of the HyFlex CM and also HyFlex EDM. These temperatures were above that of the human body. Neoniti, similar to HyFlex CM in the present study, possessed two defined peaks on heating curve and one peak on the cooling curve, showed that this instrument passed through the R-phase transformation and therefore, inherited superior properties of this intermediate phase: high fatigue resistance, narrow hysteresis [27]. Although the A_f_ temperatures of all these NiTi instruments were greater than body temperature, that showed the stable martensite phases in these instruments in the clinical situation, phase transformation of HyFlex CM Pro and Neoniti were more complex than that of Reciproc.

The transformation temperatures of the NiTi materials were one of the most important factors for mechanical properties of the rotary instruments. A transformation temperature change of the NiTi alloy, because of thermo-mechanical treatment or chemical composition variation, was utmost significant modality in altering the phase composition of the materials and consequently influencing the clinical performance of the instruments [9]. Although transformation temperatures of materials were conveniently examined by DSC analysis, other techniques, such as X-ray diffraction, metallographic examination, and scanning electronic microscopy, were useful tools to confirm the results of the DSC investigation [9].

Further research on phase transformation and compositions of the new endodontic material should be conducted.

## Conclusions

The austenite-finish (A_f_) temperatures of three NiTi instruments were higher than human body temperature, therefore, material was in the phase transformation from martensite to austenite, gives the instruments more flexibility when used in the clinical situation. The two brands, HyFlex CM Pro and Neoniti instruments, owned the intermediated R-phase transformation.

## Data Availability

The datasets used and/or analyzed during the current study are available from the corresponding author on reasonable request.

## Abbreviations

NiTi: Nickel-titanium
DSC: Differential scanning calorimeter
A_f_: Austenite-finish
A_s_: Austenite-start
M_f_: Martensite-finish
M_s_: Martensite-start
A: Austenite
M: Martensite
